# Comparison of radiosensitivity response to acute and chronic gamma irradiation in colored wheat

**DOI:** 10.1590/1678-4685-GMB-2017-0189

**Published:** 2018-06-28

**Authors:** Min Jeong Hong, Dae Yeon Kim, Joon-Woo Ahn, Si-Yong Kang, Yong Weon Seo, Jin-Baek Kim

**Affiliations:** 1 Advanced Radiation Technology Institute, Korea Atomic Energy Research Institute, Jeongeup, Republic of Korea.; 2 Division of Biotechnology, Korea University, Seoul, Republic of Korea

**Keywords:** Acute irradiation, chronic irradiation, wheat, electron spin resonance, anthocyanin

## Abstract

We aimed to investigate the biological responses induced by acute and chronic gamma irradiation in colored wheat seeds rich in natural antioxidants. After acute and chronic irradiation, the phenotypic effects on plant growth, germination rate, seedling height, and root length were examined, and the biochemical changes were investigated by analyzing the expression of antioxidant enzyme-related genes, antioxidant enzyme activities, and total antioxidant capacity. High dosages of chronic radiation reduced plant growth compared with the controls. Electron spin resonance measurement and 2,2-diphenyl-1-picrylhydrazyl activity analysis showed lower amount of free radicals in colored wheat seeds on chronic irradiation with low dosage of gamma rays compared to seeds subjected to acute irradiation. Expression levels of anthocyanin biosynthesis genes, antioxidant-related genes, and antioxidant enzyme activity in seeds and young leaves of seedling showed diverse effects in response to different dosages and types of gamma irradiation. This suggests that phenotype is affected by the dosage and type of gamma radiation, and the phytochemicals in colored wheat seeds involved in antioxidant activity to scavenge free radicals respond differently to irradiation types. This provides evidence that acute and chronic exposure to radiation have different effects on seeds and young leaves after germination.

## Introduction

Ionizing radiation has been used as a tool in plant mutation breeding programs to select for new genotypes with improved crop characteristics. Types of ionizing radiations include alpha rays, beta rays, protons, X rays, cosmic rays, gamma rays, and high-energy UV rays. These forms of radiation cause ionization, but the various types of ionizing radiation have different energy levels and can penetrate cells to different degrees. Ionizing radiation generates highly reactive oxygen species (ROS), which may cause oxidative stress. ROS, including superoxide radicals (O_2_-), hydrogen peroxide (H_2_O_2_), and hydroxyl radicals (OH), cause damage to membrane lipids (peroxidation), nucleic acids, and carbohydrates, resulting in subsequent cellular damage (Suzuki *et al.*, 2012).

To avoid oxidative stress, plants activate several protective mechanisms that control the effects of ROS on the cell (Foyer *et al.*, 1994). One of the protective systems is the enzymatic defense mechanism that operates with sequential and simultaneous actions of a number of enzymes (Foyer and Noctor, 2005). ROS accumulation is controlled by antioxidant enzymes that include a variety of scavengers, such as superoxide dismutase (SOD), catalase (CAT), ascorbate peroxidase (APX), peroxidase (POD), glutathione reductase (GR), glutathione peroxidase (GPX), dehydroascorbate reductase (DHAR), and monodehydroascorbate reductase (MDAR) (Mittler *et al.*, 2004). SOD is the first defense line against ROS and plays a critical role in scavenging superoxide (O_2_-). H_2_O_2_ is enzymatically eliminated by CAT and several POD isoforms. APX is considered the most important H_2_O_2_ scavenger, using ascorbate as the reducing agent (Gupta *et al.*, 1993, Inzé and Van Montagu, 1995). The ascorbate-glutathione cycle includes various antioxidant enzymes such as APX, DHAR, GR, and MDAR, and this pathway is involved in the scavenging of O_2_- and H_2_O_2_ (Asada, 1999). In addition, antioxidant enzymes have been shown to increase tolerance to various stresses, such as salt, drought, and heat stress that can also cause oxidative stress. Thus, antioxidant enzymes play an essential role in maintaining the balance between the generation and scavenging of ROS increased by ionizing radiation.

Phytochemicals such as phenolic compounds, flavonoids, anthocyanins, and carotenoids are believed to be effective in combating or providing protection against diseases due their antioxidant effect (Iloki-Assanga *et al.*, 2015). Colored maize (*Zea mays* L.) kernels possess a high content of phenolic compounds and anthocyanins, which have been shown to have increased free radical scavenging activity (Zilic *et al.*, 2012). Likewise, a profile of anthocyanins and other phenolics in Mexican maize also demonstrated increased free radical scavenging and reducing activities (Lopez-Martinez *et al.*, 2009). In soybean with different seed color, varieties with black and brown seeds contained large amounts of total polyphenols and anthocyanins, which provide a high content of natural antioxidants (Malencic *et al.*, 2012).

High doses of gamma rays have usually been shown to have negative effects on plant height, shape, yield, and reproductive capacity, whereas exposure to lower doses has a simulative effect. A study of *Arabidopsis* seedlings demonstrated that plants exposed to low-dose gamma irradiation (1–5 Gy) developed normally (Malencic *et al.*, 2012). Pollen fertility decreased with the increase in dose rate in rice (Kumar *et al.*, 2013), whereas in potato, low-dose gamma irradiation (2.5 Gy) led to a significant increase in the number of microtubers (Al-Safadi *et al.*, 2000). Although, radiosensitivity depends on the plant variety, harmful effects are more severe at higher doses and with higher dose rates. In addition, the type of irradiation effects can be divided into two categories according to dose intensity and periods of exposure. Acute irradiation is exposure to high doses of radiation for a short period, whereas chronic irradiation is continuous exposure to low doses of radiation over a long period.

A number of researchers have primarily studied the short-term effects of acute irradiation (El-Beltagi *et al.*, 2011; Moghaddam *et al.*, 2011; Marcu *et al.*, 2013). Kovalchuk *et al.* (2000) found that chronic irradiation caused a much higher frequency of homologous recombination (HR) compared to acute irradiation. Application of chronic radiation at 200 μGy led to a five- to six-fold increase in the frequency of HR, whereas acute irradiation (dose range of 0.1–0.5 Gy) did not increase the frequency of HR. Thus, through increased HR occurrence, chronic irradiation can produce a variety of genetic resources in plants. Several studies have been reported that effect of chronic gamma irradiation such as development of wild carrot plants, genetic variation in natural Melandrium album populations, and enzymatic hydrolysis as a pretreatment in *Brachypodium* (Kim *et al.*, 2015; Boratynski *et al.*, 2016; Karimullina *et al.*, 2016). However, there is little information on the effect of chronic irradiation on the development and oxidative defense system of plants.

The aim of this study was to investigate the biological responses induced by acute and chronic gamma exposure in colored wheat seeds, which could contain high amounts of natural antioxidants. The phenotypic effects of acute and chronic irradiation on plant growth, germination rate, seedling height, and root length were measured and the biochemical changes induced by exposing the plants to acute and chronic irradiation were investigated by analyzing the expression of antioxidant enzyme related genes, antioxidant enzyme activities, and total antioxidant capacity. The results of this study provide insights into the differing effects caused by the various dose intensities and exposure periods of gamma irradiation and provide valuable information of the ideal type of gamma irradiation for breeding programs to use as a mutagen source in crop plants.

## Materials and Methods

### Plant material and gamma irradiation

A hexaploid common wheat (*Triticum aestivum* L.) line with colored-seeds developed by Korea University (accession no. K4191) were used. Seeds of K4191 were exposed to acute and chronic gamma irradiations at dosages of 100, 300, and 500 Gy The different doses are the total absorbed dose at the end of the exposure time, which means that for the same exposure times (two weeks) different dose rates were applied to reach final dosages (100, 300, and 500 Gy - acute dose rate: 12.5 Gy/h, 37.5 Gy/h and 62.5 Gy/h; chronic dose rate: 0.298 Gy/h, 0.893 Gy/h and 1.488 Gy/h). For chronic gamma irradiation, colored wheat seeds were exposed to gamma radiation generated by a ^60^Co gamma irradiator (20 TBq of capacity, Nordion, Canada) for two weeks at 23°C in gamma phytotron room at the Korea Atomic Energy Research Institute.

For acute irradiation, colored wheat seeds were stored in a control room with identical condition of the chronic irradiation facility for two weeks and irradiated for 8 h using a ^60^Co gamma irradiator (150 TBq of capacity, AECL, Canada) in gamma irradiation facility on the last day of chronic irradiation treatment to terminate the two irradiation treatments simultaneously. Control seeds were also stored in same control room with identical condition for two weeks. After irradiation, seeds were stored at 4 °C for a short time and immediately used for further analysis.

Seeds for seedling samples were germinated for two to three days at room temperature and transferred to a Magenta box (6.5 x 6.5 x 20 cm, Greenpia Technology Inc. Seoul, Korea) containing polypro mesh as previously described (Hong *et al.,* 2012). Seedlings were grown in the Magenta box filled with 180 mL of water for 10 days in the growth facility at 23 °C and a photoperiod of 16/8 h (day/night) (Porter and Gawith, 1999). The water was freshly exchanged each day. To evaluate gamma ray sensitivity to the different doses, shoot length and root length of ten individual wheat seedlings with three biological replicates were scored by rulers. The germination assay was performed at constant temperature (25 °C) in a sterilized petri dish with water-soaked Whatman No.1 filter paper (Nakamura *et al.*, 2011). Germination rates of a total of 50 seeds with three biological replicates from each dosage and irradiation type treatment group were measured three days after imbibition.

### Electron spin resonance measurement

Non-irradiated and irradiated seeds were freeze-dried for three days in preparation for electron spin resonance (ESR) measurements. Each sample was weighed into a quartz ESR glass tube (diameter 5 mm). The glass tube was sealed with Whatman film (Whatman, GE Healthcare, Buckinghamshire, UK) and stored in a dry-oven (65 °C) at 40% ± 5% relative humidity. The ESR measurements were performed at room temperature using a JES-PX2300 (JEOL, Tokyo, Japan) X-band spectrometer equipped with a cylindrical cavity. The measurements were recorded using the following parameters: power, 0.998 mW; microwave frequency, 9.429 GHz; modulation frequency, 100 KHz; modulation width, 1 mT; magnetic center field, 337.812 mT; sweep time 30 s; time constant, 0.03 s. The ESR sample measurements were recorded simultaneously with a Mn^2+^ standard sample set in the same resonator. Variations in ESR signal intensities were measured as the peak-to-peak signal width of the first-derivative spectrum. The signal intensity was expressed in arbitrary units per unit sample weight (AU/mg).

### DPPH radical scavenging activity

The 2,2-diphenyl-1-picrylhydrazyl (DPPH)-free radical scavenging capacity of irradiated colored wheat seeds was evaluated according to the method of Blois (1958), with some modification. Samples of homogenized wheat seeds were extracted with methanol for 24 h at 4 °C. A fraction of seed extracts (0.2 mL) was added to 3.8 mL methanol solution of DPPH radical and the mixture was allowed to stand for 30 min at room temperature. The absorbance was measured at 517 nm using the UV-VIS spectrophotometer, and the inhibition of free radical DPPH was calculated using the formula:

Scavenging effect (%) = (1- *A*_sample_/*A*_control_)*100

where *A*_sample_ is the absorbance of the test compound and *A*_control_ is the absorbance of the control.

### Anthocyanin content

Total anthocyanin content was measured according to the method of Mita *et al.* (1997), with some modification. Homogenized wheat seeds were mixed with 1 mL methanol-hydrochloric acid (MeOH-HCl) (1% HCl, w/v) and incubated at 4 °C for 24 h. The absorbance was measured at 530 nm and 657 nm in a UV-VIS spectrophotometer. The anthocyanin content was determined using the formula *Q* = (*A*_530_-0.25*A*_657_) * *M* ^-1^ (*Q*: anthocyanin yield; *A*_530_ and *A*_657_: absorptions at the indicated wavelengths; *M*: mass of the plant).

### Total phenolic content

The total phenolic content was determined by a Folin-Ciocalteu assay using gallic acid (GA) as a standard Singleton *et al.* (1999). The samples were extracted with methanol (0.1 g/mL), and the mixture was prepared by mixing 0.5 mL of sample extract, 2.5 mL of 10% Folin-Ciocalteu reagents dissolved in water, and 0.75 mL of 70% Na_2_CO_3_. The mixture was incubated for 120 min at room temperature, and the absorbance was measured at 765 nm using an UV-Vis spectrophotometer (Jenway, Keison products, Chelmsford, UK). The total phenolic content was calculated as gallic acid equivalents using standard curve prepared with gallic acid solution.

### RNA extraction and qRT-PCR

Total RNA was isolated from wheat seeds and 10 day-old seedlings using Tri reagent (MRC, Cambridge, UK) according to the manufacturer’s protocol. Total RNA samples were pretreated with RNase-free DNase I to eliminate any contaminating genomic DNA. First strand cDNA was synthesized from total RNA (approximately 1 μg) using a Power cDNA synthesis kit (iNtRON Biotechnology, Gyeonggi-do, Korea). The quantitative RT-PCR reactions were performed using an Eco Real-Time PCR system (Illumina, San Diego, CA, USA). The reaction mixture (25 μL) included a SYBR premix Ex Taq II (Takara, Tokyo, Japan), the first strand cDNA, and gene-specific primers (Table 1). The two-step thermal cycling profile consisted of incubation for 10 s at 95 °C and 30 s at 65 °C. The reactions were carried out in biological triplicates using RNA samples extracted from three different plants.

**Table 1:** Primers used for gene expression analysis.

	Gene	Accession no.	Forward	Reverse
Anthocyanin-related genes	*CHS*	AB187025	CTCATGATGTATCAGCAGGG	ACATCCTTGAGGTGGAA
	*CHI*	AB187026	GCAGTACTCGGACAAGGTGA	GTTCGTTCACACCGAAACC
	*F3H*	AB187027	CCTACTTCTCGTACCCGGTG	GAACGTCGCGATCGACAG
	*DFR*	AB187028	TGCTGGAGCTTCCCGGAGC	CGTGGGGATGATGCTGATGA
	*ANS*	AB247919	GTCTCCGCGCTCTCCTTC	TCCTTCTCCTCCTCTTGAGC
	*UFGT*	GU248274	TGCCGCCGTACCTTGTGAAG	TTCCAGCCGCTGTGCGTGAA
Antioxidant-related genes	*APX*	TC22268	GCAGCTGCTGAAGGAGAAGT	CACTGGGGCCACTCACTAAT
	*CAT*	GI5711144	CCATGAGATCAAGGCCATCT	ATCTTACATGCTCGGCTTGG
	*DHAR*	GQ494009	GACCAAGGAGAACCTGATCG	CGTCGCTACTCTCACACGAC
	*GPX*	TC22467	CCCCCTGTACAAGTTCCTGA	GTCAACAACGTGACCCTCCT
	*GR*	TC84151	TGCGTCCCGAAGAAGATACT	GTTGATGTCCCCGTTGATCT
	*MDAR*	TC27229	GCTCCTCGACCATAAAGCTC	CATAGCTGCGACCAACTTGT
	*MnSOD*	EF392662	CAGAGGGTGCTGCTTTACAA	GGTCACAAGAGGGTCCTGAT
	*CuZnSOD*	U69632	CGCTCAGAGCCTCCTCTTT	CTCCTGGGGTGGGAGACAAT
Housekeeping gene	*Actin*	AB181991	GCCACACTGTTCCAATCTATGA	TGATGGAATTGTATGTCGCTTC

### Antioxidant enzyme assay

For all antioxidant enzyme assays, the proteins from 10 day-old seedling leaves were extracted by homogenizing samples with liquid nitrogen in 1 mL of 0.2 M potassium phosphate buffer (pH 7.0) containing 0.1 mM ethylenediaminetetraacetic acid (EDTA) at 4 °C. Total protein content was determined by the Bradford assay using BSA as a standard (Bradford, 1976). APX activity was measured according to Nakano and Asada (1981). CAT activity was measured following the method of Aebi (1984). POD activity was assayed according to the method of Kwak *et al.* (1995) using pyrogallol as a substrate. SOD activity was determined by measuring the inhibition of the photochemical reduction of Nitro Blue Tetrazolium (NBT) using the method of Giannopolitis and Ries (1977).

### Measurement of chlorophyll content

To determine the content of chlorophyll and carotenoids, samples of homogenized 10 day-old wheat seedlings were suspended in 100% acetone at 4 °C in the dark (Lichtenthaler and Buschmann, 2001). The homogenized samples were centrifuged at 12,000 x *g* for 10 min and the supernatant was used for pigment determination. The absorbance of the supernatant was recorded at 470, 644.8, and 661.6 nm using a UV/VIS spectrophotometer (Jenway, Keison Products, Chelmsford, UK). The concentration of chlorophyll content was estimated using extinction coefficients provided by Lichtenthaler (1987).

### Statistical analysis

To test for normality, we used an Anderson-Darling test. The statistical analyses were performed with MINITAB 16 software. The statistical significances of differences between mean values were determined using the Student’s *t*-test. Differences in the mean values were considered significant when the *p*-value was less than 0.05.

## Results

### Effect of acute and chronic radiation treatments on plant growth

**Figure 1:**
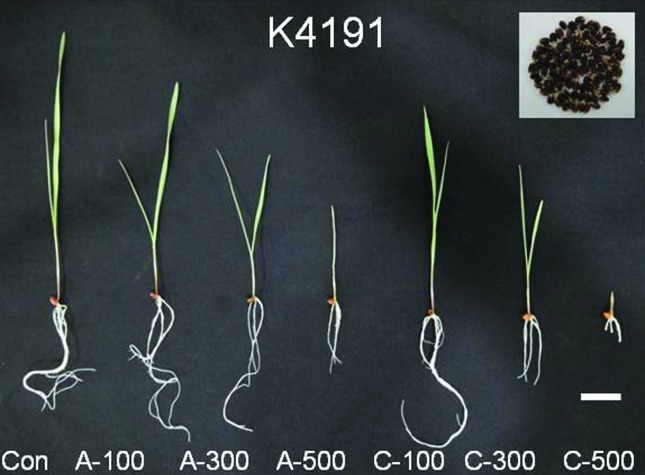
Phenotypic effect of gamma irradiation in colored wheat seeds (K4191). Con: control (non-irradiated samples); A: acute irradiation; C: chronic irradiation; 100, 300, and 500: gamma irradiation dose. Scale bar: 2 cm.

The correlation between the types of gamma irradiation and germination rate and other morphological traits is presented in Table 2. Following acute and chronic irradiation at doses of 100, 300, and 500 Gy, the germination rate of colored wheat seed was not significantly different between the control and irradiated seeds. Yet shoot and root length was significantly reduced by treatment with the higher doses of both acute and chronic gamma radiation (Figure 1 and Table 2). Chronic irradiation caused the most severe growth inhibition compared with the other treatment and control.

**Table 2:** Germination and plant growth in two wheat varieties exposed to different radiation doses. Values shown are means ± SD for *n* = 3 independent experiments.

Radiation exposure method	Dose (Gy)	Germination (%)	Shoot length	Root length
	Control	98.3	13.97 ± 1.24	10.87 ± 1.13
Acute	100	98.3	10.13 ± 0.72	9.4 ± 0.95
	300	93.3	7.03 ± 1.04	6.50 ± 1.04
	500	96.7	4.04 ± 1.03	2.62 ± 0.67
Chronic	100	93.3	11.03 ± 0.65	11.38 ± 1.25
	300	95.0	8.26 ± 0.66	5.46 ± 0.93
	500	98.3	1.77 ± 0.24	1.03 ± 0.31

#### Measurement of total antioxidant capacity in seeds

**Figure 2:**
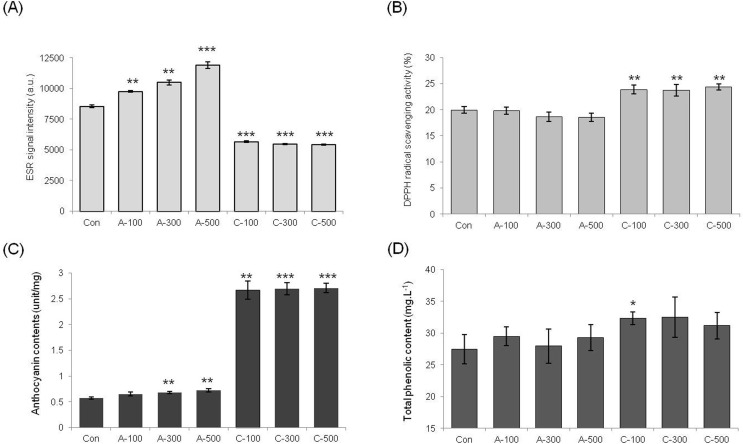
Effect of acute and chronic gamma irradiation on wheat dry seeds. (A) Free radical content after irradiation of wheat dry seeds assessed by ESR spectroscopy. (B) Effects of acute and chronic gamma irradiation on DPPH radical scavenging activity in wheat dry seeds. (C) Anthocyanin content after acute and chronic gamma irradiation in wheat dry seeds. (D) Comparison of the total phenolic content of wheat dry seeds in response to acute and chronic gamma irradiation. Con: control (non-irradiated seeds); A: acute irradiation; C: chronic irradiation; 100, 300, and 500: gamma irradiation dose. Each bar represents mean ± SD for *n* = 3 independent experiments.

The ESR method was used to measure superoxide radical scavenging activities directly in the irradiated wheat seeds. Interestingly, the ESR signal of chronically irradiated seeds was lower than that of non-irradiated seeds (Figure 2A). These results further indicate that lower amounts of free radicals were detected when chronically irradiated for two weeks with a low dosage of gamma rays than in seeds treated with acute irradiation. The intensity of the ESR signals increased linearly with the increase in the absorbed dose in the case of the plants treated with acute irradiation, which means acute irradiation induced more severe oxidative damage. To validate this result, free radical scavenging activities were analyzed by the measurement of DPPH scavenging ratio to further compare the radiation effects of the different types of gamma ray treatment (Figure 2B). The DPPH radical scavenging activities were slightly increased in all dosages of chronic irradiation. Anthocyanin and total phenolic contents were also measured to show the effect of phytochemicals in colored wheat seeds on antioxidant capacity under different gamma irradiation type and dosage. Higher levels of anthocyanin were found in all dosages of colored wheat seeds treated with chronic irradiation than in the control condition and acute irradiated seeds (Figure 2C). Additionally, a small increase in total phenol content was detected in chronic irradiated seeds (Figure 2D).

#### Expression of anthocyanin and antioxidant biosynthesis related genes in seeds

**Figure 3:**
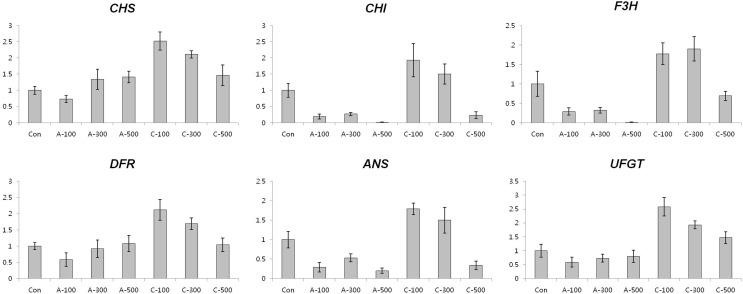
Expression profiling of anthocyanin biosynthesis genes in colored wheat dry seeds. *CHS*; *chalcone synthase*, *CHI*; *chalcone isomerase*, *F3H*; flavanone 3-hydorxylase, *DFR*; *dihydroflavonol 4-reductase*, *ANS*; *anthocyanidin synthase*, *UFGT*; *UDP-glucose: flavonoid 3-O-glucosyltransferase*. A: acute irradiation; C: chronic irradiation; 100, 300, and 500: gamma irradiation dose. Each bar represents mean ± SD for *n* = 3 independent experiments.

**Figure 4:**
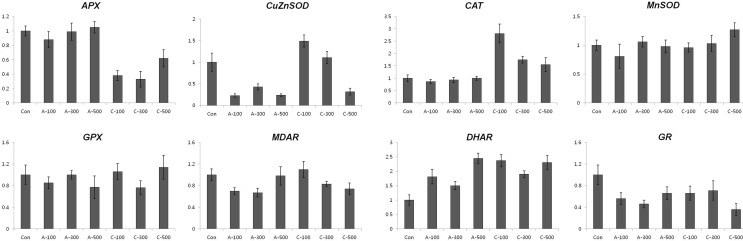
Expression profiling of antioxidant biosynthesis related genes in colored wheat dry seeds. *APX*; *ascorbate peroxidase*, *CuZnSOD*; *CuZn superoxide dismutase*, *CAT*; *catalase*, *MnSOD*; *Mn superoxide dismutase*, *GPX*; *glutathione peroxidase*, *MDAR*; *monodehydroascorbate reductase*, *DHAR*; *dehydroascorbate reductase*, *GR*; *glutathione reductase*. A: acute irradiation; C: chronic irradiation; 100, 300, and 500: gamma irradiation dose. Each bar represents mean ± SD for *n* = 3 independent experiments.

To test whether the accumulation of anthocyanins following chronic gamma irradiation of colored wheat seeds was accompanied by an increase in the level of transcripts corresponding to anthocyanin biosynthesis genes, we investigated the expression patterns of anthocyanin biosynthesis genes using qRT-PCR. Generally, the level of transcripts of anthocyanin biosynthesis related genes such as *CHS*, *CHI*, *F3H*, *DFR*, *ANS*, and *UFGT* were found to be higher in all dosages of chronically irradiated seeds than in seeds under the other conditions (Figure 3), corresponding with the anthocyanin content results shown in Figure 2C. Expression of the *APX* transcript was reduced in seeds by both acute and chronic gamma irradiation (Figure 4).

*CAT* and *ZnCuSOD* transcripts were expressed at the maximal level at 100 Gy of chronic gamma irradiated wheat seeds and then consistently declined with increased gamma radiation dosages. The *MnSOD* transcript level slightly increased at 500 Gy after chronic gamma irradiation and *GPX* and *MDAR* transcripts showed similar or decreased levels compared to the control following both acute and chronic gamma irradiation. Lastly, *GR* transcripts were slightly down-regulated after acute and chronic gamma irradiation.

#### Antioxidant enzyme activity in seeds

**Figure 5:**
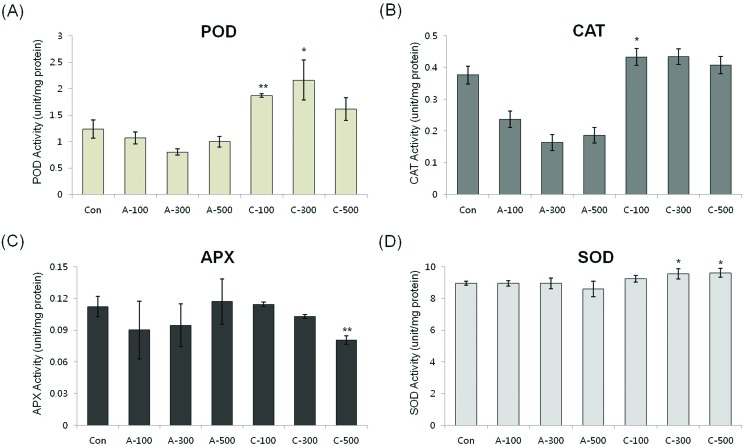
Effect of acute and chronic gamma irradiation treatment on the activities of (A) POD, (B) CAT, (C) APX, and (D) SOD in colored wheat dry seeds. A: acute irradiation; C: chronic irradiation; 100, 300, and 500: gamma irradiation dose. Each bar represents mean ± SD for *n* = 3 independent experiments.

In order to determine the antioxidant responses of wheat seeds to acute and chronic gamma irradiation, we measured the enzymatic activity of POD, CAT, APX, and SOD in the two types of gamma irradiated wheat seeds. POD activity was higher following all dosage of chronic irradiation. CAT activity in seeds treated with chronic irradiation were similar to control seeds, however the activity was reduced in seeds treated with all dosage of acute irradiation. APX activity was slightly decreased with increasing dose of chronic irradiation and SOD activity did not change significantly between non-irradiated seeds and the two different irradiation types of gamma rays (Figure 5).

#### Measurement of total antioxidant capacity in seedlings

**Figure 6:**
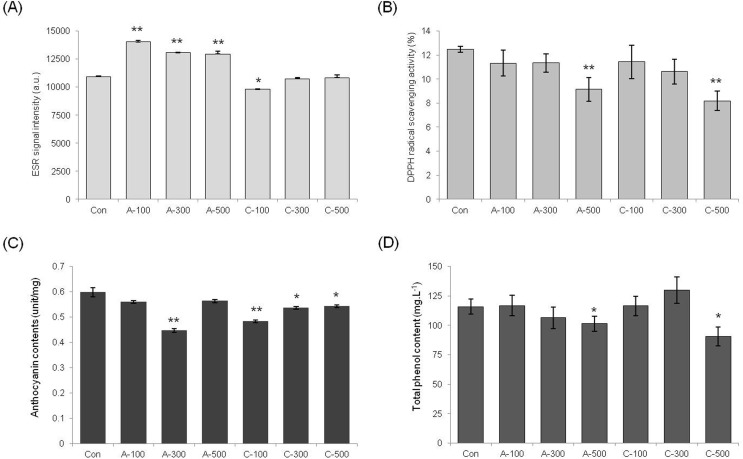
Effect of acute and chronic gamma irradiation on wheat seedlings. (A) Free radical content after irradiation of wheat seedlings assessed by ESR spectroscopy. (B) Effects of acute and chronic gamma irradiation on DPPH radical scavenging activity in wheat seedlings. (C) Anthocyanin content after acute and chronic gamma irradiation in wheat seedlings. (D) Comparison of the total phenolic content of wheat seedlings in response to acute and chronic gamma irradiation. Con: control (non-irradiated samples); A: acute irradiation; C: chronic irradiation; 100, 300, and 500: gamma irradiation dose. Each bar represents mean ± SD for *n* = 3 independent experiments.

The seeds irradiated with acute and chronic radiation were germinated to compare total antioxidant capacity in seedling. Interestingly, even though the intensity of the ESR signal was slightly increased in acute irradiation, DPPH radical scavenging activities were similar in both acute and chronic irradiation treatments (Figure 6A,B). Total anthocyanin content of chronically irradiated plants did not increase and even measured at lower levels than the control plants (Figure 6C). In addition, the total phenol content was not significantly different between acute and chronic irradiation treatments (Figure 6D). Therefore, the results from germinated seedlings were not consistent with those from the seeds following the two different type of gamma irradiation treatment.

#### Expression of anthocyanin and antioxidant biosynthesis related genes in seedlings

**Figure 7:**
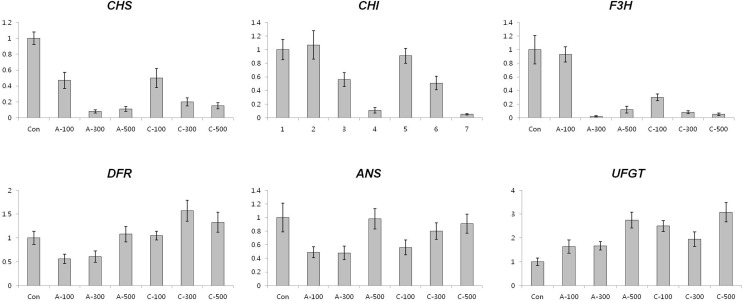
Expression profiling of anthocyanin biosynthesis genes in wheat seedlings. *CHS*, *CHI*, *F3H*, *DFR*, *ANS*, and *UFGT*. A: acute irradiation; C: chronic irradiation; 100, 300, and 500: gamma irradiation dose. Each bar represents mean ± SD for *n* = 3 independent experiments.

**Figure 8:**
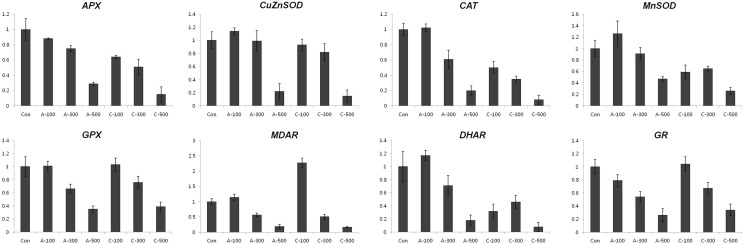
Expression profiling of antioxidant biosynthesis related genes in wheat seedlings. *APX*, *CuZnSOD*, *CAT*, *MnSOD*, *GPX*, *MDAR*, *DHAR*, and *GR*. A: acute irradiation; C: chronic irradiation; 100, 300, and 500: gamma irradiation dose. Each bar represents mean ± SD for *n* = 3 independent experiments.

In wheat seedlings, the amount of transcripts of *CHS* was decreased in all the radiation conditions (Figure 7). Except for the transcripts of *ANS* following 300 Gy and 500 Gy of chronic irradiation, the expression of anthocyanin biosynthesis genes was reduced compared with control plants. Interestingly, transcripts of *UFGT* were increased in all dosages of the two different irradiation types, The expression levels of antioxidant-related genes, such as *APX*, *CAT*, *CuZnSOD*, *MnSOD,* and *DHAR* in plants subjected to acute irradiation were slightly higher than in those subjected to chronic irradiation (Figure 8). The transcript level of *MDAR* in 100 Gy of chronic irradiated plants was 2-fold up-regulated compared with control plants.

#### Antioxidant enzyme activity in seedlings

**Figure 9:**
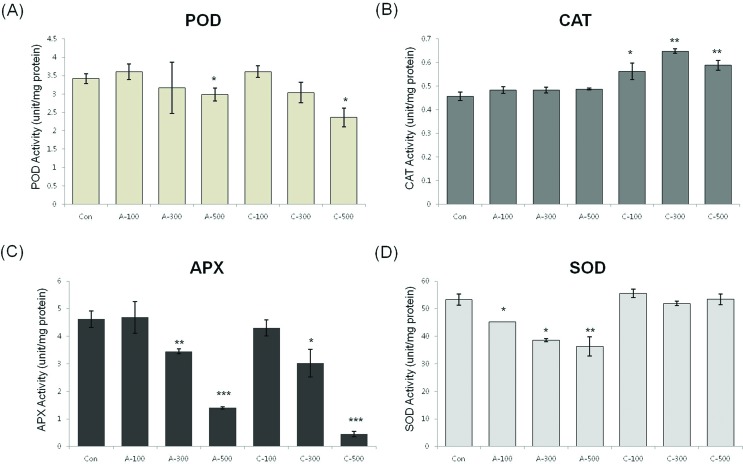
Effect of acute and chronic gamma irradiation treatment on the activities of (A) POD, (B) CAT, (C) APX, and (D) SOD in wheat seedlings. Con: control (non-irradiated samples); A: acute irradiation; C: chronic irradiation; 100, 300, and 500: gamma irradiation dose. Each bar represents Mean ± SD for average *n* = 3 independent experiments.

In order to determine the antioxidant responses of wheat seedlings to acute and chronic gamma irradiation, we measured the enzymatic activity of SOD, CAT, POD, and APX in wheat seedlings treated by two different irradiation types. POD activity in both treatments showed a similar pattern and CAT activity was increased in chronic irradiation (Figure 9A,B). The APX activity in colored wheat seeds was decreased following increased dose in both acute and chronic irradiation treatment and SOD activity was higher in seedlings following chronic irradiation (Figure 9C,D).

#### Chlorophyll content

**Figure 10:**
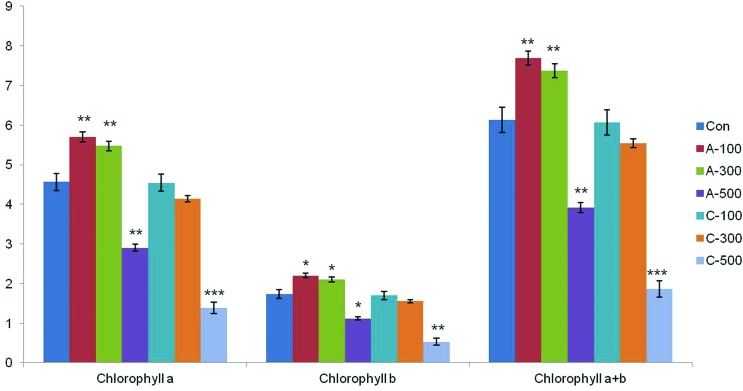
Effect of gamma radiation doses on chlorophyll *a*, chlorophyll *b*, and total chlorophyll content in wheat seedlings. Con: control (non-irradiated samples); A: acute irradiation; C: chronic irradiation; 100, 300, and 500: gamma irradiation dose. Each bar represents mean ± SD for *n* = 3 independent experiments.

The contents of chlorophyll *a*, chlorophyll *b*, and total chlorophyll were determined for both the control and irradiated samples. Among the two irradiation types, acute irradiation caused relatively higher damage to chlorophyll content (Figure 10). Interestingly, the content of chlorophyll *a* and *b* were most increased under the condition of 100 Gy and 300 Gy of acute irradiation.

### Discussion

Gamma radiation is used in crop mutation breeding programs to improve qualitative and quantitative characters of many crop species. Gamma rays are a more economical and effective tool compared to other ionizing radiation types because of their availability and penetration capacity. Generally, free radicals generated by ionization can have negative effects on plant germination, growth, morphology, and yield (Mittler, 2002). Additionally, stress signals and responses triggered by high doses of gamma irradiation have harmful effects on the physiological and biochemical traits of plants (Aly and El-Beltagi, 2010; Jan *et al.*, 2012). However, in some cases, exposure to low levels of ionizing radiation is known to have a beneficial effect on plant growth, which is referred to as hormesis (Calabrese, 2002). The objective of this study was to evaluate the biological responses induced by acute and chronic gamma irradiation in colored wheat seeds. Currently, most studies have been conducted and focused on the evaluation of the biological response to acute irradiation, whereas relatively few studies investigated the physiological response to chronic irradiation even though it has been shown that exposure to chronic gamma irradiation generates lower amounts of free radicals in comparison to acute irradiation in plants (Kovalchuk *et al.*, 2000; Vandenhove *et al.*, 2010; Hong *et al.*, 2014).

Our study indicates that acute and chronic irradiation of wheat may have different effects on plant growth and oxidative stress responses. Within gamma irradiation type treatment groups, the growth of plants treated with high doses of chronic irradiation was more reduced and caused severe growth inhibition compared with the controls (Figure 1, Table 2). Free radical molecules are induced by oxidative damage caused by various biotic and abiotic stresses (Suzuki *et al.*, 2012; Kocsy *et al.*, 2013). Gamma rays can induce free radicals, and these molecules are strong oxidative stress factors that damage lipids, proteins, and DNA within plant cells (Shikazono *et al.*, 2005; Moghaddam *et al.*, 2011). These observations indicate that treatment with high doses of gamma irradiation has harmful effects on plant growth and development through the increase of free radicals. However, interestingly, the ESR signal of chronically irradiated colored wheat seeds was lower than that of non-irradiated seeds, and the DPPH radical scavenging activities were slightly increased in wheat treated with all dosage of chronic irradiation compared with both control and acute irradiated seeds (Figure 2A,B).

When plants are exposed to gamma irradiation, free radical concentrations increase with increasing absorbed doses (Rana *et al.*, 2010; Marcu *et al.*, 2013). Flavonoids and polyphenols are compounds that protect cells against the oxidative effects of ROS (Gill and Tuteja, 2010; Dangles, 2012). Anthocyanins are flavonoids, which are believed to be related to the overall antioxidant capacity of the plant. Anthocyanins function as major scavengers to eliminate free radicals (Tsuda *et al.*, 2000; Radovanovic and Radovanovic, 2010). ESR signals showed that under chronic gamma irradiation conditions the wheat seeds had lower free radical content than acute gamma irradiation (Figure 2C and Figure 3). The anthocyanin contents were significantly higher in all dosages of chronically irradiated seeds than in acute-irradiated seeds, and high anthocyanin content following chronic irradiation could increase the capacity for free radical scavenging. Therefore, even though the seeds under the chronic irradiation condition were damaged more severely that after acute irradiation, it is supposed that phytochemicals such as anthocyanins in the colored wheat seeds affected the regulation of free radical contents caused by gamma irradiation. These results demonstrate that the antioxidant activity of anthocyanins is related to their ability for scavenging of free radicals.

Excessive ROS levels may cause cell injury and death because they can generate bimolecular oxidative stress (Tan *et al.*, 1998; Poljsak *et al.*, 2013). During an acute or chronic exposure to ionizing radiation, protective mechanisms like antioxidant enzymes are activated to scavenge for the ROS molecules (Foyer *et al.*, 1994). Several studies provided proof of enhanced activities of antioxidant enzymes following gamma irradiation (Kovalchuk *et al.*, 2000; Foyer and Noctor, 2005; Aly and El-Beltagi, 2010). Therefore, the expression of enzymatic antioxidant genes including *SOD*, *CAT*, *APX*, *MDAR*, *DHAR*, *GR*, and *GPX* and the activity of antioxidant enzymes such as POD, CAT, APX, and SOD were measured to compare the enzymatic antioxidants of the two irradiation types of dry seeds. Transcripts of two genes, *CAT* and *CuZnSOD,* were expressed at the maximal level after 100 Gy of chronic gamma irradiation and showed higher expression levels compared with control and acute irradiated dry seeds (Figure 4). In addition, POD activity was higher in all dosage of chronic irradiated dry seeds, and CAT activity in dry seeds treated with chronic irradiation was higher than in acute irradiated dry seeds (Figure 5). Although our results show that gene expression cannot be directly correlated with enzyme activity, various functions of antioxidant enzymes and their transcriptional regulation may still be involved in the cellular protection against ROS from both the environment and metabolism.

In young leaves of seedlings, low ESR signals were detected in chronically irradiated plants; High content of formed anthocyanin through chronic gamma irradiation in dry seeds may be involved in the compensatory mechanisms of inhibition of free radicals when wheat seedling growth. (Figures 2 and 6). But, the patterns of anthocyanin and phenolic contents in seedlings did not correlate with those in dry seeds, and the transcription levels of anthocyanin biosynthesis genes, antioxidant biosynthesis related genes, and antioxidant enzyme activities in seedlings did not show significant differences between the different type of gamma irradiation other than in a dosage dependent manner (Figure 7, 8 and 9). This result is important from the aspect of showing that the stimulatory effect of acute and chronic gamma irradiation doses for dry seeds and seedling growth may not be the same.

A previous study observed that chlorophyll content increased with increasing exposure to 100 Gy of gamma radiation in wheat (Borzouei *et al.*, 2010). In lettuce, the level of photosynthetic pigments (chlorophyll *a*, chlorophyll *b*, carotenoids) increased at doses ranging from 2–30 Gy, whereas at higher doses (up to 70 Gy) it decreased (Marcu *et al.*, 2013). Irradiation of red pepper (16 Gy) and lupine (20 Gy) resulted in a significant increase in the total chlorophyll content (Khodary and Moussa, 2003; Kim *et al.*, 2005). In the current study, seeds irradiated with acute gamma doses ranging from 100 to 300 Gy had enhanced photosynthetic pigment content, but the chlorophyll content declined following chronic irradiation (Figure 10). This indicates that the type of gamma irradiation differently affects chlorophyll contents and degradation. High doses of gamma irradiation cause a reduction in photosynthesis due to the disturbance of chlorophyll biosynthesis or degradation, concomitant with a loss of photosynthetic capacity (Strid *et al.*, 1990; Dale *et al.*, 1997; Kim *et al.*, 2011). Chloroplasts are more susceptible to gamma irradiation than other organelles (Wi *et al.*, 2007).

Most studies on the effects of radiation have investigated the high-acute external exposure on the plants. To our knowledge, our study is the first attempt that compares the effects of chronic and acute gamma irradiation in wheat. After colored wheat seeds were irradiated with acute and chronic gamma radiation at various doses (100, 300, and 500 Gy), their growth pattern inversely correlated with the gamma dose and was inhibited at higher doses. Although colored wheat seeds were treated with the same dose of irradiation with different type of exposure, remarkably plant growth was differently affected depending on type of gamma irradiation exposure. Chronic gamma irradiation caused severe damage and plant growth inhibition to wheat to a much greater extent than acute irradiation. However, the anthocyanin content in colored wheat seeds after chronic irradiation treatment was significantly higher than in the control.

Generally, high levels of free radicals can lead to the overproduction of ROS in plants, which ultimately results in oxidative stress. Increasing the content of free radicals through increased radiation dose may be related to the reduction in plant growth. Increasing the anthocyanin content can serve as a mechanism to regulate free radical contents. Therefore, it is supposed that phytochemicals in colored seed wheat exposed to chronic radiation treatment could affect total antioxidant capacity, such as ESR signal, free radical scavenging capacity, and antioxidant enzyme activities. In addition, radiosensitivity of wheat seeds depends on both the irradiation type, acute or chronic irradiation. Our study provides a valuable basis for further research on gamma-irradiation mediated mutagenesis in wheat.
